# Enhanced accuracy of cardiac ^82^Rb PET functional parameters with 16-compared to 8-gated imaging on a long-axial-field-of-view PET system: validation against echocardiography

**DOI:** 10.1007/s00259-026-07892-5

**Published:** 2026-04-16

**Authors:** Nasir Gözlügöl, Foroud Aghapour Zangeneh, Hasan Sari, Wolfram A. Bosbach, Konstantinos Zeimpekis, Axel Rominger, Christoph Gräni, Federico Caobelli

**Affiliations:** 1 Department of Nuclear Medicine, Inselspital, Bern University Hospital, University of Bern, Freiburgstrasse 18, Bern, 3010 Switzerland; 2 Siemens Healthineers International AG, Zurich, Switzerland; 3 Department of Cardiology, Inselspital, Bern University Hospital, University of Bern, Bern, Switzerland

**Keywords:** ^82^Rb-PET/CT, 16-gates PET protocol, LAFOV PET, Cardiac imaging, Coronary artery disease

## Abstract

**Introduction:**

Left ventricular (LV) ejection fraction (EF) and volumes derived from ECG-gated ^82^Rb PET are clinically relevant, but their assessment can be impaired due to limited temporal sampling and low count statistics. Long axial field-of-view (LAFOV) PET/CT systems provide substantially higher temporal resolution compared to standard, analog PET systems, which allows to yield higher sensitivity without the need for increasing tracer activity. We aimed to evaluate, whether increasing ECG gating from 8 to 16 frames on ^82^Rb LAFOV PET improves the accuracy of LV functional parameters. To that end, we used transthoracic echocardiography (TTE) as reference of standard.

**Materials and methods:**

We retrospectively included 124 consecutive patients undergoing clinically indicated rest–stress ^82^Rb PET/CT on a LAFOV system. All patients received 370 MBq ^82^Rb for both rest and stress acquisitions. Rest images were reconstructed using both 8- and 16-gated frames. PET-derived LVEF, end-diastolic volume (EDV), and end-systolic volume (ESV) were compared with TTE values obtained within a median of 26 days. Agreement was assessed using Wilcoxon test, Pearson correlation, and Bland–Altman analysis.

**Results:**

Mean rest LVEF was 52.3 ± 15.5% with 8-gated PET, 58.1 ± 13.5% with 16-gated PET, and 57.7 ± 9.7% on TTE. LVEF derived from 16-gated PET showed no significant difference compared with TTE (*p* = 0.24), with fair correlation (*r* = 0.58) and minimal bias (+ 1.13%). In contrast, 8-gated PET significantly underestimated LVEF compared with TTE (mean bias − 5.49%, *p* < 0.001; *r* = 0.38), particularly at lower LVEF values. EDV was comparable between 8- and 16-gated PET but showed modest overestimation versus TTE for both reconstructions. For ESV, 8-gated PET significantly overestimated values compared with TTE (*p* = 0.002), whereas 16-gated PET showed improved agreement with no statistically significant difference (*p* = 0.085).

**Conclusion:**

On LAFOV PET/CT, increasing temporal resolution from 8 to 16 ECG-gated frames significantly improves the accuracy of LV functional assessment, particularly for ESV and LVEF, yielding close agreement with TTE. These improvements were achieved using low ^82^Rb activities, supporting the routine clinical implementation of 16-gated protocols on LAFOV systems to enhance functional cardiac assessment without increasing radiation burden.

**Supplementary Information:**

The online version contains supplementary material available at https://doi.org/10.1007/s00259-026-07892-5.

## Introduction

 In patients with suspected or known coronary artery disease (CAD), the assessment of left ventricular (LV) ejection fraction (LVEF) and LV volumes is recommended as part of clinical care, given their impact on risk stratification and management [1, 2]. Positron emission tomography (PET) with e.g. ^82^Rb-chloride is currently embedded in international guidelines in the assessment of CAD [3], as it provides robust qualitative and quantitative information on myocardial perfusion, while also allowing the assessment of LV functional parameters including LVEF, end-diastolic volume (EDV) and end-systolic volume (ESV) [4, 5]. However, the reliability of functional data derived from electrocardiogram (ECG)-gated ^82^Rb-PET imaging critically depends on count statistics and temporal sampling. Low count statistics, especially under constraint of low tracer activity, the number of ECG-gated frames used for temporal sampling and suboptimal gating quality can lead to inaccurate estimation of LV volumes and EF [6, 7].

In conventional PET with standard axial field-of-view (SAFOV), ECG-gated acquisitions typically employ 8 ECG-gated frames per cardiac cycle to balance temporal resolution against image noise [8]. While this approach provides a reasonable estimation of LV function, it may smooth over the true systolic and diastolic extrema, leading to under- or overestimation of EDV, ESV, and consequently inaccurate LVEF assessment [9]. Increasing the number of frames, e.g., from 8 to 16, could enhance the accuracy of these measurements by offering finer temporal sampling of the cardiac cycle [10]. Yet, the major limitation of 16-gate reconstruction is reduced counts per frame, which can degrade image quality and introduce bias in small, count-limited datasets [11].

This trade-off between temporal and count resolution has discouraged the routine use of 16-gate protocols in ^82^Rb cardiac PET [12]. The short half-life of ^82^Rb (76s) requires that relatively high activities be administered, and further increase to achieve adequate counts statistics for higher gated frames number would cause higher patient radiation exposure [13, 14]. Therefore, most clinical centers continue to use 8-gated acquisitions as the practical standard for LV functional analysis [15].

To date, dedicated studies using ⁸²Rb on LAFOV PET/CT that directly compare 8- versus 16-gated reconstructions are lacking, whereas prior work has mainly addressed this question on SAFOV PET or PET/MR platforms [7, 16]. This knowledge gap has become particularly relevant with the introduction of long axial field-of-view (LAFOV) PET/CT systems, which provide a four- to sixfold increase in sensitivity compared with conventional SAFOV PET systems [17–19]. The resulting improvement in count statistics at low administered activities makes high-temporal-resolution cardiac imaging more feasible without increasing patient and staff dose [20], and recent data suggest that LAFOV PET may improve signal-to-noise ratio and quantitative stability across gated frames [21, 22]. Therefore, in the present study, we aimed to evaluate whether a 16-gate protocol on a LAFOV PET/CT system improves the accuracy of LV functional parameters compared with the standard 8-gated approach, using TTE as the reference modality.

## Materials and methods

### Study population

This retrospective study included consecutive patients who underwent ^82^Rb cardiac PET/CT for the evaluation of myocardial ischemia between January and July 2025 as part of clinical care. Pharmacologic stress was performed using Regadenoson 400 µg (*n* = 107) or Dobutamine (*n* = 17), as summarized in Table 1. All patients underwent routine TTE performed within a median of 26 days from the PET examination (range 2–37 days). Patients with incomplete ECG gating, rhythm disturbances impairing reliable ECG gating and functional analysis, such as atrial fibrillation/flutter or frequent premature beats, left bundle branch block or missing echocardiographic data were excluded. No patient was excluded because of contouring failure on gated PET analysis. The study was approved by the local ethics committee and was conducted in accordance with the Declaration of Helsinki.

**Table 1:** Clinical and demographic characteristics of included patients. CAD = coronary artery disease. WMA= wall motion abnormalities

Patient characteristics	
Number of patients	124
Age (yrs)	67.9 ± 12.5
Male (%)	84 (67.7%)
CAD (n)	44 (35.5%)
Patients with WMA	18 (14.5%)
Heart rate at rest (bpm)	67.4 ± 11.6
Heart rate at stress (bpm)	98.5 ± 25.5
Beta-blocker use	49 (39.5%)
Calcium channel antagonist use	27 (21.8%)
Any rate-controlling medication	61 (49.2%)
Stress agent	
Regadenoson	107 (86.3%)
Dobutamine	17 (13.7%)

### Imaging protocol

All scans were performed on a LAFOV PET/CT system (Biograph Vision Quadra, Siemens Healthineers, Knoxville, TN, USA). Each patient underwent a rest/stress protocol with 2 × 370 MBq ^82^Rb-chloride, eluted from a CardioGen-82 radionuclide generator (Bracco Imaging S.p.A, Milan, Italy). The radiotracer was automatically infused through an antecubital vein within approximately 20 s. Both at rest and under stress, images were acquired in list-mode over 7 min, with simultaneous ECG gating. For each image set, images were reconstructed using both 8- and 16-gated frames per cardiac cycle.

Each dataset was reconstructed with a proprietary ordered-subset expectation maximization (OSEM) algorithm, applying attenuation and scatter correction derived from a low-dose CT scan as previously published [23]. For the analysis of LV functional values, rest images were compared with TTE, whereas stress images were additionally analyzed for comparison between 8- and 16-gated PET reconstructions. PET datasets were analyzed independently, and the readers were blinded to the echocardiographic results.

In an exploratory subsample of 10 patients, image-based signal-to-noise ratio (SNR) was assessed on reconstructed PET datasets by relating the mean myocardial signal to the standard deviation of background activity for both 8- and 16-gated reconstructions at rest and stress.

### Transthoracic echocardiography

TTE values were used as the reference standard for comparison with PET-derived LV function and was performed by the referring cardiologists as part of the patients’ routine cardiac work-up. EDV, ESV and LVEF were quantified according to the biplane Simpson method.

### Statistical analysis

All statistical analyses were performed using Python (version 3.11) with the libraries pandas, NumPy, SciPy, and matplotlib. Continuous variables are expressed as mean ± standard deviation (SD) and/or as median and interquartile range (IQR). Comparisons of Left-ventricular ejection fraction, end-diastolic volume, and end-systolic volume among 8-gate frames PET, 16-gate frames PET, and TTE were evaluated using Wilcoxon test. Agreement between modalities was assessed by Bland–Altman analysis, and correlations between PET- and TTE-derived parameters were quantified using the Pearson correlation coefficient (R). Statistical equivalence between modalities was further assessed by calculating an interclass correlation coefficient (*κ*). *p*-value < 0.05 was considered statistically significant.

For the LVEF transition analysis, categories were based on Echocardiography classification of LV systolic function [24], in which severely impaired LVEF is defined as ≤ 35%, impaired LVEF as 36–49%, borderline low LVEF as 50–54%, and normal LVEF as ≥ 55%. For the purpose of the alluvial plot, the BSE borderline low and normal groups were merged into a single ≥ 50% category to improve interpretability and avoid sparse transition cells.

## Results

**Fig. 1:**
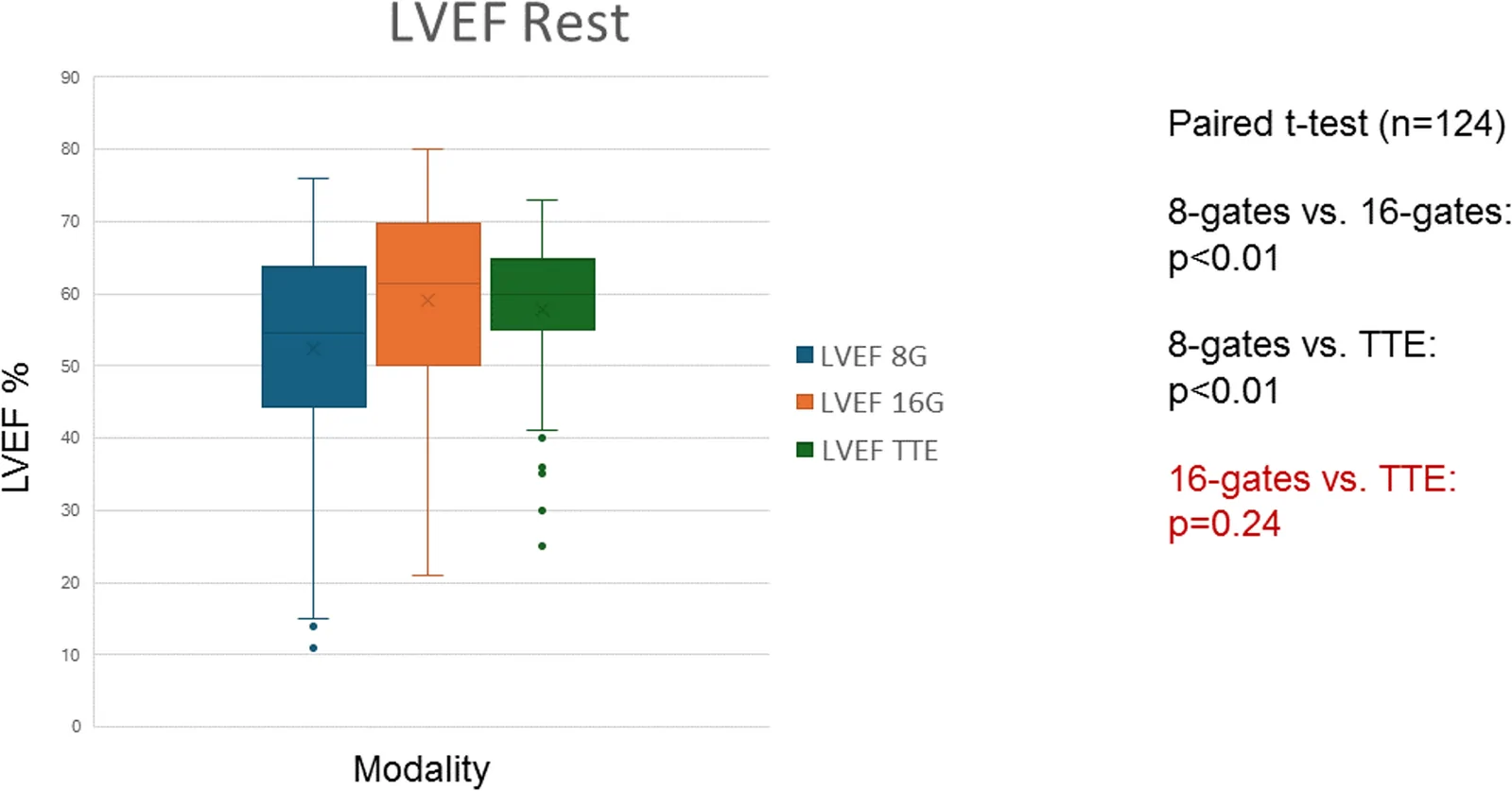
Left ventricular ejection fraction (LVEF) at rest. Boxplots depicting LVEF from 8-gate PET, 16-gate PET, and transthoracic echocardiography (TTE)

One hundred and twenty-four patients were eventually considered in the study analysis (84 males, 67.7%, age 68.2 ± 12,9 years). Regional wall motion abnormalities (hypokinesia or akinesia) were present in 18/124 patients (14.5%) and were visually detectable on both 8- and 16-gated PET reconstructions. Mean ± SD and median [interquartile range] of rest and stress LVEF, EDV and ESV derived from 8- and 16-gated PET reconstructions and TTE are summarized in Table 2. Table 3 summarizes the results of the comparison between modalities both at stress and rest. No significant difference was observed between 16-gate frames PET and TTE (*p* = 0.24, Fig. 1). Conversely, LVEF values were different between 8-gate frames and 16-gate frames PET imaging (*p* < 0.01) and between 8-gate frames PET and TTE (*p* < 0.01).

**Table 2:** Functional parameters assessed by 8-gate-PET, 16-gate PET and Echocardiography

Imaging parameter	8-gate PET mean (SD)	8-gate PET median (IQR)	16-gate PET mean (SD)	16-gate PET median (IQR)	TTE mean (SD)	TTE median (IQR)
LVEF (%) rest	52.32 (15.50)	54.50 (44.75–63.25)	58.94 (13.08)	61.50 (50.00-69.25)	57.81 (8.74)	60.00 (55.00-64.25)
EDV (mL) rest	116.37 (46.16)	110.50 (81.75–143.00)	115.93 (39.20)	114.00 (86.00-138.75)	105.05 (36.35)	95.50 (80.50–127.00)
ESV (mL) rest	58.73 (39.77)	49.50 (30.00-74.25)	50.32 (31.77)	46.00 (26.00-63.75)	45.55 (20.34)	42.50 (31.00–54.00)
LVEF (%) stress	59.45 (14.60)	63.00 (53.00-70.25)	65.85 (13.43)	69.00 (60.75-74.00)	-	-
EDV (mL) stress	107.16 (42.10)	103.50 (79.00-129.00)	115.06 (41.46)	114.50 (87.00-138.25)	-	-
ESV (mL) stress	47.00 (31.65)	38.50 (24.00–58.00)	44.30 (32.76)	36.00 (22.75–54.25)	-	-

**Table 3:** Comparisons between modalities for both stress and rest PET. ICC = intraclass correlation coefficient. Stress TTE comparisons were not available

Imaging parameter	8-gate PET vs. 16-gate PET				8-gate PET vs. TTE				16-gate PET vs. TTE			
	*p* value (Wilcoxon test)	Regression analysis		Interclass coefficient k	*p* value (Wilcoxon test)	Regression analysis		Interclass coefficient k	*p* value (Wilcoxon test)	Regression analysis		Interclass coefficient k
		Pearsoncoefficient(*R*)	*p* value			Pearsoncoefficient(*R*)	*p* value			Pearsoncoefficient(*R*)	*p* value	
LVEF (%) rest	< 0.01	0.708	< 0.001	0.633	< 0.01	0.384	< 0.001	0.301	0.240	0.584	< 0.001	0.539
EDV (mL) rest	0.09	0.657	< 0.001	0.603	0.040	0.515	< 0.001	0.487	0.020	0.604	< 0.001	0.582
ESV (mL) rest	0.001	0.568	< 0.001	0.483	0.002	0.603	< 0.001	0.454	0.085	0.758	< 0.001	0.681
LVEF (%) stress	< 0.01	0.740	< 0.001	0.777	-	-	-	-	-	-	-	-
EDV (mL) stress	< 0.01	0.898	< 0.001	0.931	-	-	-	-	-	-	-	-
ESV (mL) stress	0.002	0.988	< 0.001	0.950	-	-	-	-	-	-	-	-

**Fig. 2:**
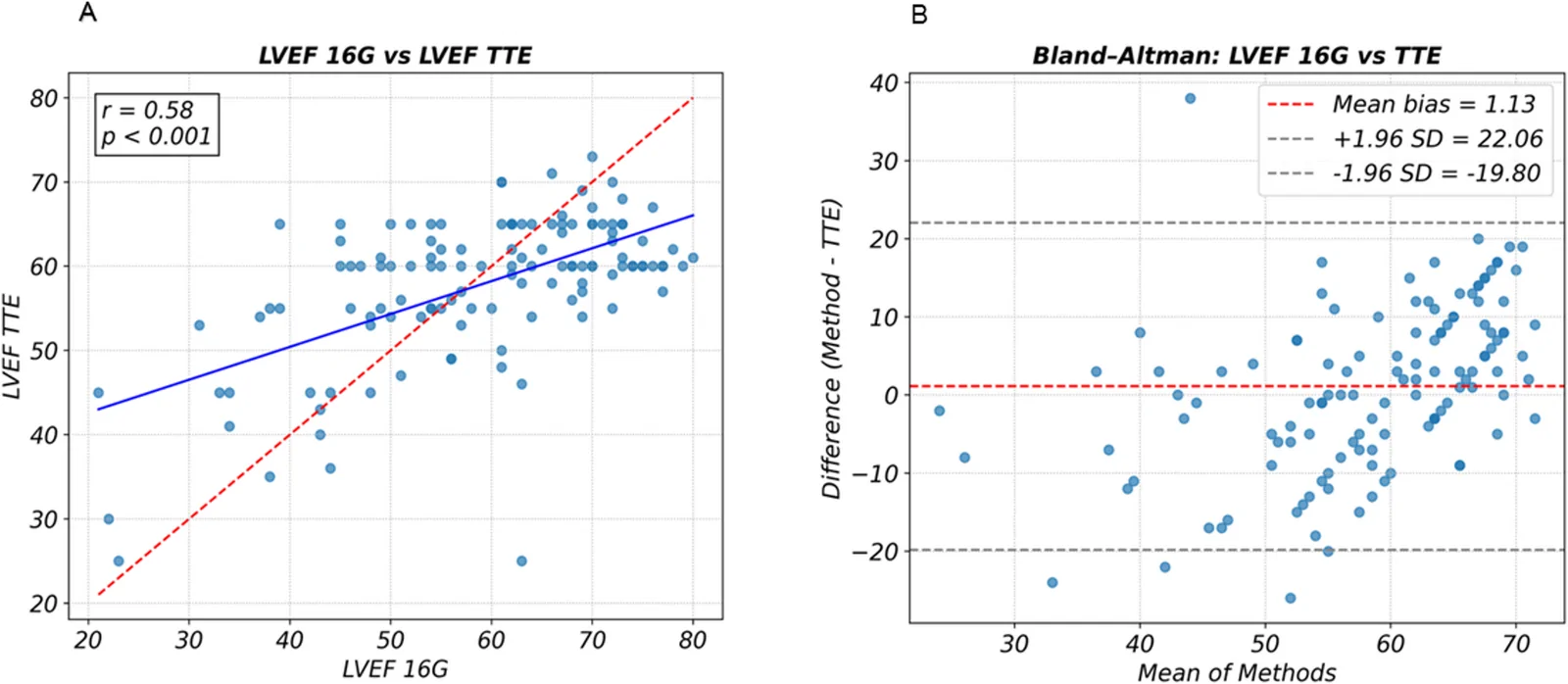
Comparison of left ventricular ejection fraction (LVEF) measurements between 16-gate PET and transthoracic echocardiography (TTE). The left panel (**A**) shows a scatter plot illustrating the relationship between LVEF obtained with 16-gate PET and TTE. The right panel (**B**) displays the corresponding Bland–Altman plot depicting the agreement between both methods

**Fig. 3:**
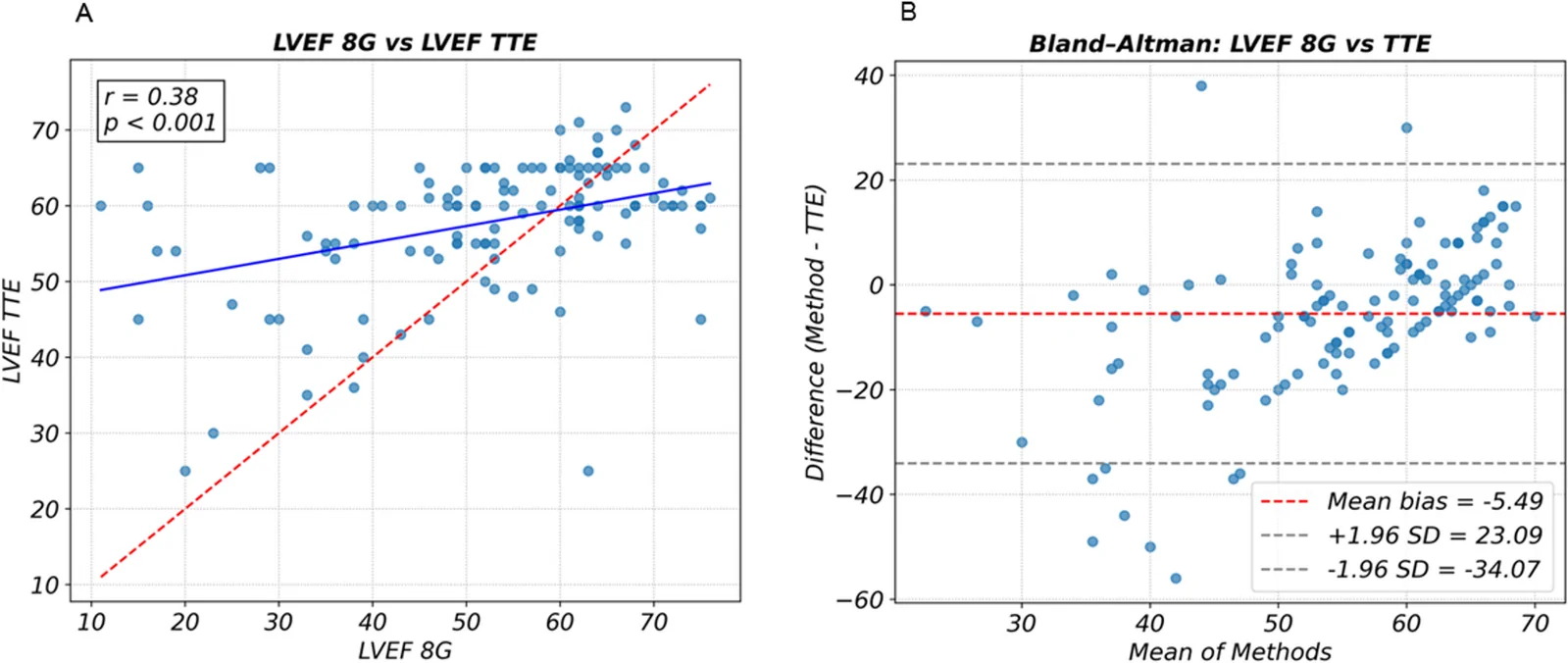
Comparison of left ventricular ejection fraction (LVEF) measurements between 8-gate PET and transthoracic echocardiography (TTE). The left panel (**A**) shows a scatter plot of LVEF values derived from 8-gate PET and TTE. The right panel (**B**) displays the corresponding Bland–Altman plot illustrating the agreement between the two methods

Specifically, LVEF values derived from 16-gate frames PET imaging showed a fair correlation with TTE (*r* = 0.58, *p* < 0.001) (Fig. 2A), with a substantial agreement on Bland–Altman analysis (limits of agreement − 19.80 to 22.06, mean bias + 1.13% [95% CI -0.77 to + 3.03], Fig. 2B). Conversely, 8-gate frames PET images had a weaker correlation with TTE images (*r* = 0.38, *p* = 0.01, Fig. 3A) with a systematic LVEF underestimation (limits of agreement − 34.07 to 23.09, mean bias of − 5.49% [95% CI -8.08 to -2.90], Fig. 3B). Of note, the underestimation tended to be more pronounced for lower LVEF values.

**Fig. 4:**
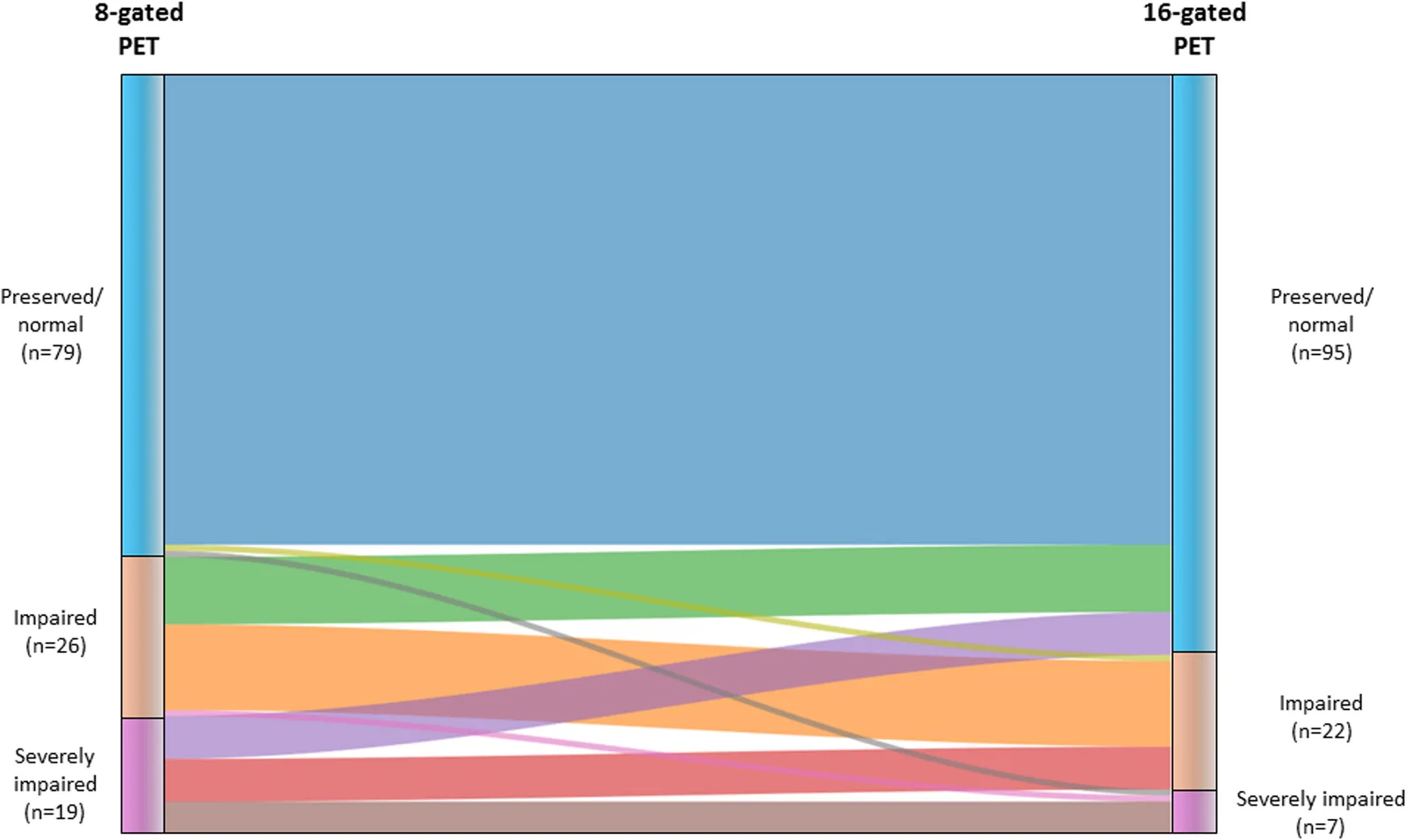
Alluvial plot showing transitions in left ventricular ejection fraction (LVEF) categories between 8-gate and 16-gate measurements (*n* = 124). Individual LVEF values were classified as Normal (≥ 50%), Impaired (36–49%), or severly impaired (≤ 35%). Ribbon width is proportional to the number of patients moving between categories, and side boxes represent the category distribution at each time point. Blue: Normal to Normal (*n* = 77); Orange: Impaired to Impaired (*n* = 14); Green: Impaired to Normal (*n* = 11); Red: Severely impaired to Impaired (*n* = 7); Purple: Severely impaired to Normal (*n* = 7); Brown: Severely impaired to Severely impaired (*n* = 5); Pink: Impaired to severely impaired (*n* = 1); Gray: Normal to Severely impaired (*n* = 1); Olive green: Normal to Impaired (*n* = 1)

When LVEF was categorized as normal/preserved (≥ 50%), impaired (36–49%), or severely impaired (≤ 35%), 16-gated PET classified more patients as having normal LV function than 8-gated PET (95/124 [76.6%] vs. 79/124 [63.7%]). Among the 79 patients classified as normal on 8-gated PET, 77 (97.5%) remained normal on 16-gated PET, whereas 2 (2.5%) were reclassified to a lower category. In contrast, among patients with reduced LVEF on 8-gated PET, reclassification toward a higher category was frequent: 11/26 (42.3%) patients classified as impaired and 7/19 (36.8%) classified as severely impaired were reclassified as normal on 16-gated PET. Overall, worsening of category from 8-gated to 16-gated PET was uncommon and occurred in only 2/124 patients (1.6%) (Fig. 4). A sensitivity analysis according to the absolute PET–TTE interval confirmed a systematic underestimation of LVEF by 8-gated PET compared with echocardiography across all time windows examined. Mean bias was − 5.49% in the overall cohort, -4.19% for PET–TTE intervals ≤ 21 days, -4.23% for intervals ≤ 14 days, and − 3.24% for intervals ≤ 7 days. The distribution of PET–TTE delays is shown in Supplemental Fig. 1, and the corresponding mean bias values are summarized in Supplemental Table 1.

**Fig. 5:**
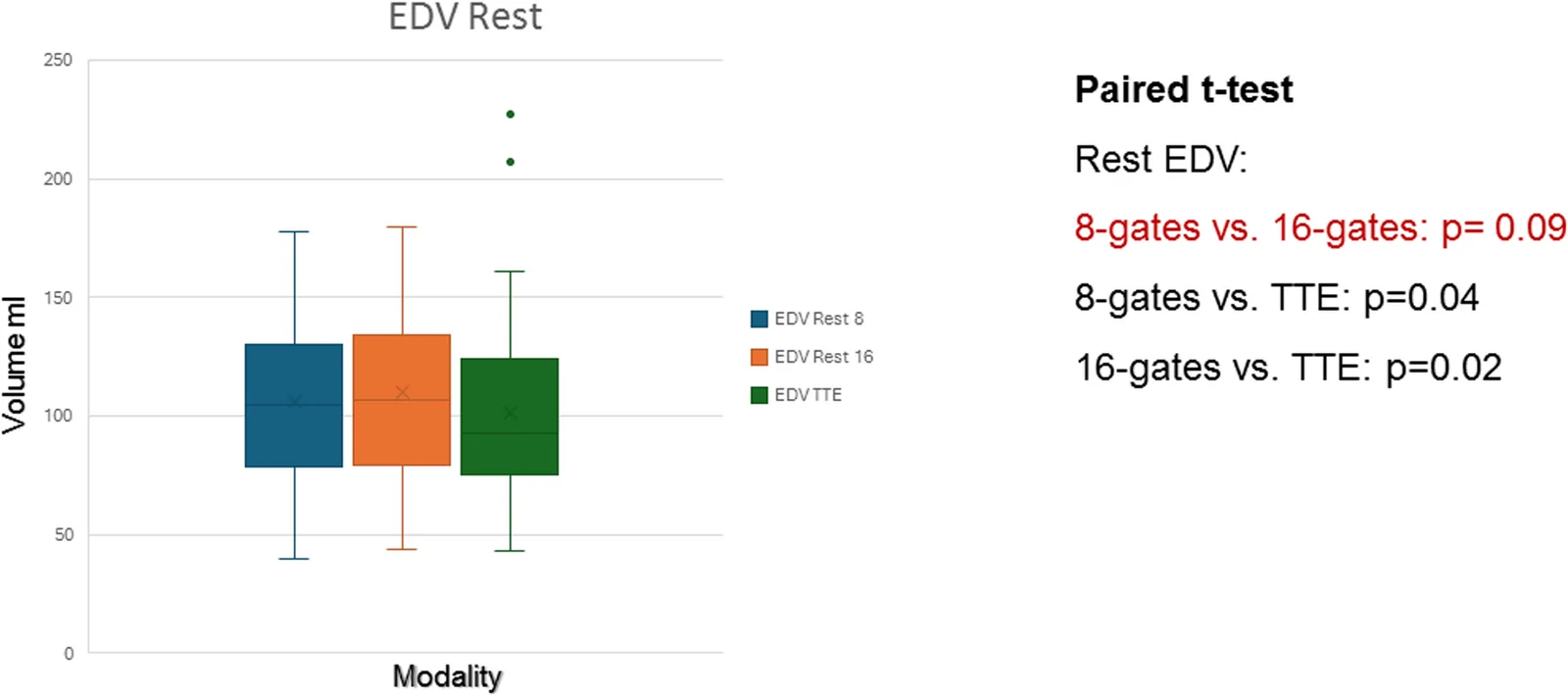
End-diastolic volume (EDV) at rest. Boxplots comparing EDV from 8-gate PET, 16-gate PET, and TTE

EDV values were comparable between 8-gate frames and 16-gate frames PET imaging (Fig. 5), but showed differences both when comparing TTE to 8-gate frames PET (*p* = 0.04), and 16-gate frames PET (*p* = 0.02).

**Fig. 6:**
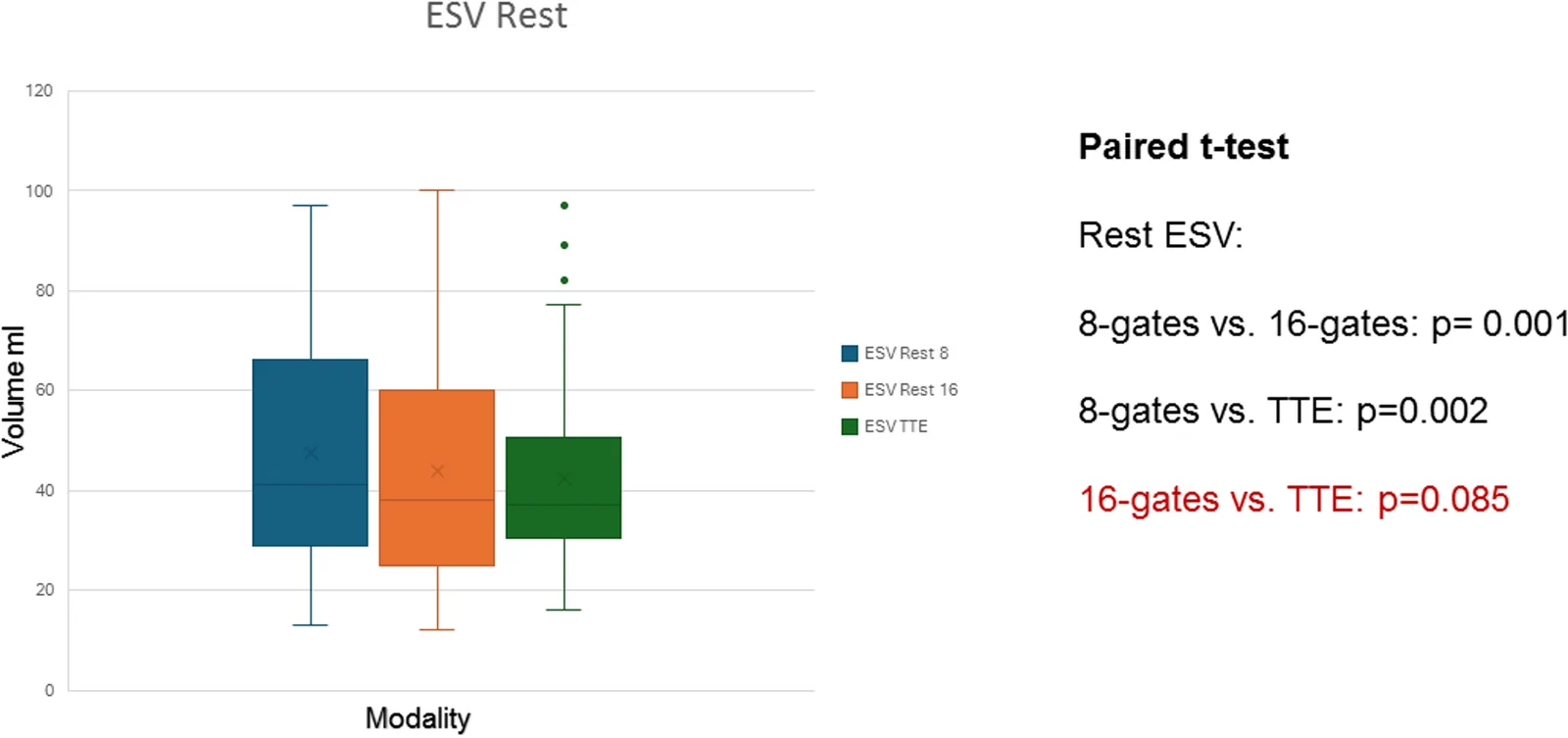
End-systolic volume (ESV) at rest. Boxplots comparing ESV from 8-gate PET, 16-gate PET, and TTE

Considering ESV, there was a significant difference between 8-gate frames and 16-gate frames PET (Fig. 6, *p* = 0.001), being values derived from 8-gate frames PET higher. To note, values from 8-gate frames were different from those on TTE (*p* = 0.002), whereas values from 16-gate frames PET were not (*p* = 0.085).

Stress LV functional analysis was additionally performed in all 124 patients. Mean stress LVEF was 59.45 ± 14.60% with 8-gated PET and 65.85 ± 13.43% with 16-gated PET (*p* < 0.01 and *R* = 0.74). Mean stress EDV was 107.16 ± 42.10 mL and 115.06 ± 41.46 mL, respectively (*p* < 0.01 and *R* = 0.898), whereas mean stress ESV was 47.00 ± 31.65 mL with 8-gated PET and 44.30 ± 32.76 mL with 16-gated PET (*p* = 0.002 and *R* = 0.988). Bland-Altman analysis showed a mean difference of -6.40% for stress LVEF (limits of agreement from − 21.09 to 8.30, 95% CI -7.73 to -5.06). For stress EDV, the mean difference was − 7.90 mL (limits of agreement from − 34.47 to 18.66, 95% CI -10.31 to -5.49), while for stress ESV, the mean difference was 2.70 mL (limits of agreement from − 16.59 to 22.00, 95% CI 0.95 to 4.45, Supplemental Fig. 2). No relevant differences in image quality or LV functional parameters were observed between patients undergoing regadenoson and dobutamine stress.

In an exploratory image-quality analysis performed in 10 patients, image-based SNR was consistently lower for 16-gated than for 8-gated reconstructions, both at rest (7.12 ± 1.56 vs. 8.99 ± 2.05) and during stress (7.41 ± 1.72 vs. 9.27 ± 2.36), corresponding to an average reduction of approximately 20%; detailed per-patient data are provided in Supplemental Table 2.

## Discussion

In this single-center cohort of 124 consecutive patients undergoing clinical ^82^Rb cardiac PET on a LAFOV PET/CT system, increasing the temporal sampling from 8 to 16 ECG-gate frames significantly improved the agreement of LV functional parameters with TTE. Of note, 8-gate frames PET systematically underestimated LVEF and this underestimation was more pronounced at lower LVEF values, whereas 16-gate frames PET showed no significant difference from TTE and only a minimal residual bias. A sensitivity analysis according to the absolute PET–TTE interval showed that this systematic underestimation with 8-gated PET was present across all examined time windows, although it was slightly less pronounced at shorter intervals. This supports the robustness of the finding and suggests that the observed underestimation is not solely attributable to temporal separation between PET and echocardiography. These findings indicate that, on a LAFOV system, 16-frame gating can largely correct the tendency of low-frame protocols to underestimate LVEF.

The observation that 8-gated PET underestimates LVEF, especially in patients with impaired systolic function, is consistent with previous studies on conventional PET and SPECT systems, where limited temporal sampling has been shown to distort the identification of true end-systolic and end-diastolic phases [6–10, 25–27]. Accurate quantification of LVEF is clinically important, because LVEF and LV volumes remain central determinants for risk stratification and treatment decisions, including device therapy and advanced heart failure management [1–5, 28]. The transition analysis suggests that LVEF classification is broadly consistent between 8-gated PET and 16-gated PET for patients with normal function, but that 16-gate PET tends to yield higher LVEF estimates in patients with abnormal function, resulting in frequent reclassification from impaired or severely impaired to normal. This pattern is compatible with reduced gating-related underestimation and improved temporal sampling at 16 gates, and it indicates that the choice of gating scheme can materially affect categorical interpretation of LVEF, particularly around clinically relevant thresholds. By bringing PET-derived LVEF much closer to the echocardiographic reference, the 16-gated protocol increases the reliability of LV functional information obtained during routine perfusion imaging.

Our findings are broadly consistent with prior work showing that PET-derived LV functional parameters are influenced by the chosen ECG-gating strategy, but they also highlight important differences related to study population and imaging platform. In a study of young healthy subjects, Lassen et al. [7] found similar rest and stress LVEF estimates and repeatability for 8- and 16-gate ^82^Rb PET, suggesting that both approaches may be interchangeable when only volumetric assessment is required. In contrast, in our clinical cohort with a broader range of LV function, 8-gate PET systematically underestimated LVEF, whereas 16-gate PET showed substantially closer agreement with TTE, indicating that the benefit of higher temporal sampling becomes more relevant in routine patients, particularly when systolic function is reduced. Likewise, Villagran Asiares et al. [16] demonstrated in a PET/MR cross-validation study that cardiac functional estimates are sensitive to the applied ECG-gating method, with high correlations but still substantial disagreement versus MRI. Our data are in line with this general concept and extend it by showing that on ^82^Rb LAFOV PET/CT, increasing temporal sampling from 8 to 16 gates improves agreement with the clinical reference standard, especially for LVEF and ESV.

The analysis of LV volumes helps to understand the mechanism behind these differences. EDV values were comparable between 8- and 16-gate frames PET and showed only modest overestimation compared with TTE. Such small discrepancies are in line with previously reported inter-modality differences between tomographic techniques and echocardiography and are usually of limited clinical relevance [15, 29, 30]. This relative robustness of EDV to the chosen number of gates is physiologically plausible, because diastole occupies a longer portion of the cardiac cycle and the true end-diastolic phase is therefore less sensitive to temporal undersampling [30, 31].

In contrast, ESV was clearly affected by the gating strategy. In our cohort, 8-gate PET significantly overestimated ESV, while 16-gate PET markedly reduced this discrepancy and the remaining difference was no longer statistically significant. LVEF is calculated as LVEF (%) = [(EDV - ESV) / EDV] × 100. Therefore, an overestimation of ESV, even in the presence of only minor changes in EDV, directly leads to an underestimation of LVEF. The shorter duration of systole, the smallest volume with consequent partial volume effect and the sharper curvature of the volume–time curve around end-systole make the exact localization of this point highly dependent on temporal resolution [25–27, 30, 31]. The improved alignment of ESV between 16-gate PET and TTE in our study therefore provides a mechanistic explanation for the more accurate LVEF obtained with the 16-gate protocol.

To date, the main argument against using 16 gates in clinical practice has been the trade-off between temporal resolution and counts per frame. With analog, SAFOV PET systems, increasing the number of frames can increase image noise and reduce the robustness of automatic contour detection, especially in smaller ventricles or low-dose datasets [11, 12]. This has contributed to the widespread adoption of 8-gate protocols as a pragmatic compromise between accuracy and image quality [11, 12, 15]. LAFOV PET changes this balance, allowing shorter reconstructed frames without a substantial loss of image quality due to increased sensitivity [16–18, 21–23, 32–35]. Of note, all acquisitions in our study were performed with a relatively low administered activity of 370 MBq ^82^Rb, yet the 16-gated reconstructions yielded stable functional parameters that closely matched TTE. In fact, the fixed activity of 370 MBq in our study was substantially lower than the 740–1480 MBq activity range recommended in the EANM procedural guideline for conventional 3D ^82^Rb PET [36], with fixed doses of 740–1110 MBq considered acceptable depending on scanner sensitivity, thereby providing context for the low-dose advantage of the LAFOV approach. This supports the concept that high-temporal-resolution ECG-gating can be implemented on LAFOV systems without increasing tracer dose.

Several recent publications have characterized LAFOV and total-body PET systems in terms of sensitivity, image quality and dosimetry and have illustrated their potential for ultra-low-dose and shortened myocardial perfusion protocols [16–23, 32–35]. However, systematic evaluations of how this improved hardware performance should translate into optimized functional PET protocols are still limited. Our study expands on this knowledge, by specifically comparing 8- and 16-gate reconstructions in a real-world cohort of patients referred for clinically indicated ^82^Rb LAFOV PET.

TTE was chosen as the reference modality because it is widely available, guideline-endorsed and routinely used for LV functional assessment in patients with suspected or known coronary artery disease [1–4, 37]. Although cardiac magnetic resonance (CMR) imaging is widely regarded as the gold standard for volumetric assessment, TTE represents the real-world comparator for PET-derived functional parameters in most clinical pathways. In this context, the finding that 16-gate PET essentially removes the systematic bias of 8-gate PET against TTE is clinically relevant, as it may reduce the need for additional TTE studies performed solely to confirm LVEF after PET.

Overall, our results support the routine use of 16-gate reconstructions for ^82^Rb cardiac PET on LAFOV PET systems. By providing ESV and LVEF values that are substantially closer to TTE than those derived from 8-gate protocols, 16-gate PET improves the reliability of LV functional assessment that accompanies myocardial perfusion imaging and may help to streamline patient management, particularly in those near clinically relevant LVEF thresholds.

### Limitations

This study has several limitations. First, it was retrospective and single-centre, using a standard reconstruction protocol and a single software solution for functional analysis. The results may therefore not be directly generalized to other vendors, reconstruction settings or analysis tools, although the physical principles behind the effect of temporal sampling on LV function quantification are likely to be similar. Further, the percentage of rejected cardiac cycles was not available retrospectively from the archived clinical reconstruction data and could therefore not be analyzed.

Second, TTE was used as the reference standard rather than CMR. While TTE reflects routine clinical practice and guideline-based workflows [1–4, 37], CMR is considered the gold standard for volumetric assessment [38]. Furthermore, TTE is subject to inter- and intra-observer variability and methodological heterogeneity, which can contribute to apparent discrepancies when it is used as a reference for validating PET-derived parameters [39, 40]. These factors may have influenced the magnitude of the observed agreement between PET and TTE.

Third, TTE and PET studies were not performed on the same day but within a clinically acceptable time window. Interim changes in loading conditions, rhythm or medical therapy cannot be fully excluded, especially in patients with unstable clinical status, and may have introduced additional variability.

Fourth, no systematic follow up echocardiographic or clinical outcome data were available in this retrospective cohort. Therefore, although 16-gate PET resulted in substantial reclassification of LVEF categories and showed better agreement with echocardiography, the longitudinal and clinical implications of this reclassification cannot be determined from the present study. Future prospective studies should assess whether these differences translate into clinically meaningful changes in risk stratification or patient management.

In addition, no formal interrater reproducibility analysis was performed for either PET or TTE measurements, and therefore observer-related variability in image interpretation could not be assessed directly in this study.

Finally, we focused on global LV functional parameters, and did not focus on regional wall motion or more advanced functional markers such as strain, which are increasingly available with both TTE and PET [4, 29, 37]. Whether 16-gate frames LAFOV PET also improves the accuracy and reproducibility of these more sophisticated indices compared with 8-gate protocols remains to be addressed in future prospective studies.

## Conclusion

In patients undergoing clinical ⁸²Rb cardiac PET on a LAFOV system, increasing temporal resolution from 8 to 16 ECG-gate frames markedly improved the accuracy of LV functional parameters, with 16-gate reconstructions showing close agreement with transthoracic echocardiography, particularly for LVEF and ESV. This was achieved using low activities of 370 MBq for both rest and stress, indicating that high-temporal-resolution gating is feasible without increasing dose or scan time. These findings support routine use of 16-gate protocols for ⁸²Rb cardiac PET on LAFOV systems to enhance the reliability of LV functional assessment in everyday practice.

## Supplementary Information

Below is the link to the electronic supplementary material.

Supplementary Material 1

Supplementary Material 2

Supplementary Material 3

Supplementary Material 4

## Data Availability

The datasets generated during the current study are available from the corresponding author on reasonable request.
