# Supplementary material for: Enhanced accuracy of cardiac 82Rb PET functional parameters with 16-compared to 8-gated imaging on a long-axial-field-of-view PET system: validation against echocardiography
Source: Eur J Nucl Med Mol Imaging. 2026 Apr 16;53(7):4718–28. doi: 10.1007/s00259-026-07892-5 (PMC13649810; doi:10.1007/s00259-026-07892-5)
Supplement: Supplementary file 1 [file 259_2026_7892_MOESM1_ESM.docx]

| **Subset** | **n** | **Bias PET-TTE**  **LVEF** | **Mean absolut error** | **Pearson r** | **ICC** |
| --- | --- | --- | --- | --- | --- |
| All | 124 | -5.49 | 10.96 | 0.38 | 0.30 |
| ≤21 days | 42 | -4.19 | 9.33 | 0.51 | 0.45 |
| ≤14 days | 31 | -4.23 | 7.19 | 0.65 | 0.57 |
| ≤7 days | 17 | -3.24 | 6.06 | 0.85 | 0.79 |

**Supplemental Table 1**: Sensitivity analysis depending on time interval between PET and Echocardiography (TTE), depicting the Bias and correlation between left ventricular ejection Fraction (LVEF) as calculated on PET and TTE. ICC= interclass coefficient.
