# Supplementary material for: Enhanced accuracy of cardiac 82Rb PET functional parameters with 16-compared to 8-gated imaging on a long-axial-field-of-view PET system: validation against echocardiography
Source: Eur J Nucl Med Mol Imaging. 2026 Apr 16;53(7):4718–28. doi: 10.1007/s00259-026-07892-5 (PMC13649810; doi:10.1007/s00259-026-07892-5)
Supplement: Supplementary file 2 [file 259_2026_7892_MOESM2_ESM.docx]

|  | **BG SD** | | | | **Cardiac Mean** | | | | **SNR** | | | |
| --- | --- | --- | --- | --- | --- | --- | --- | --- | --- | --- | --- | --- |
|  | REST 8 | REST 16 | STRESS 8 | STRESS 16 | REST 8 | REST 16 | STRESS 8 | STRESS 16 | REST 8 | REST 16 | STRESS 8 | STRESS 16 |
|  | 2.7 | 3.6 | 2.8 | 4.0 | 27.5 | 27.2 | 36.7 | 36.0 | 10.3 | 7.6 | 13.2 | 9.0 |
|  | 2.2 | 2.7 | 2.9 | 3.2 | 30.4 | 29.9 | 32.3 | 30.1 | 13.8 | 11.2 | 11.3 | 9.5 |
|  | 2.45 | 3.18 | 2.68 | 3.52 | 20.54 | 20.57 | 22.46 | 23.51 | 8.4 | 6.5 | 8.4 | 6.7 |
|  | 3.44 | 3.62 | 4.48 | 4.59 | 25.05 | 24.72 | 26.33 | 25.93 | 7.3 | 6.8 | 5.9 | 5.6 |
|  | 2.95 | 3.56 | 3.63 | 4.56 | 21.3 | 20.98 | 26.13 | 25.89 | 7.2 | 5.9 | 7.2 | 5.7 |
|  | 2.75 | 3.24 | 3.41 | 3.79 | 22.52 | 22.4 | 38 | 37.58 | 8.2 | 6.9 | 11.1 | 9.9 |
|  | 2.51 | 3.58 | 3.15 | 4 | 24.82 | 24.38 | 32.72 | 32.7 | 9.9 | 6.8 | 10.4 | 8.2 |
|  | 2.65 | 3.27 | 2.59 | 3.39 | 21.28 | 21.19 | 27.36 | 27.25 | 8.0 | 6.5 | 10.6 | 8.0 |
|  | 3.22 | 4.11 | 3.34 | 4.61 | 22.92 | 22.82 | 23.97 | 24.85 | 7.1 | 5.6 | 7.2 | 5.4 |
|  | 2.17 | 2.81 | 3.43 | 4.08 | 20.99 | 20.66 | 25.53 | 24.83 | 9.7 | 7.4 | 7.4 | 6.1 |
| Mean | 2.7 | 3.4 | 3.2 | 4.0 | 23.7 | 23.5 | 29.2 | 28.9 | 9.0 | 7.1 | 9.3 | 7.4 |
| SD | 0.41 | 0.42 | 0.57 | 0.51 | 3.23 | 3.11 | 5.41 | 4.99 | 2.05 | 1.57 | 2.38 | 1.72 |

**Supplemental Table 2**. Analysis in a subset of 10 patients regarding signal-to-noise ratio. BG SD = Background standard deviation, SNR= signal-to-noise Ratio. Rest 8 = rest PET, 8 gated. Rest 16 = rest PET, 16-gated; Stress 8 = stress PET, 8-gated; Stress 16= stress PET, 16-gated
